# Enhanced Efficiency and Stability of Sky Blue Perovskite Light-Emitting Diodes via Introducing Lead Acetate

**DOI:** 10.3390/molecules29112425

**Published:** 2024-05-21

**Authors:** Zequan Zhang, Qiaoli Niu, Baoxiang Chai, Junhao Xiong, Yuqing Chen, Wenjin Zeng, Xinwen Peng, Emmanuel Iheanyichukwu Iwuoha, Ruidong Xia

**Affiliations:** 1 State Key Laboratory of Organic Electronics and Information Displays & Institute of Advanced Materials (IAM), Nanjing University of Posts & Telecommunications, 9 Wenyuan Road, Nanjing 210023, China; zzq15235813322@163.com (Z.Z.); 18300633693@163.com (B.C.); 18790401377@163.com (J.X.); 1023061507@njupt.edu.cn (Y.C.); iamwjzeng@njupt.edu.cn (W.Z.); 2 State Key Laboratory of Pulp and Paper Engineering, School of Light Industry and Engineering, South China University of Technology, Guangzhou 510640, China; fexwpeng@scut.edu.cn; 3 Sensor Lab (University of the Western Cape Sensor Laboratories), 4th Floor Chemical Sciences Building, University of the Western Cape, Robert Sobukwe Road, Bellville, Cape Town 7535, South Africa; eiwuoha@uwc.ac.za

**Keywords:** perovskite light-emitting diodes, Pb(Ac)_2_, defect passivation

## Abstract

All-inorganic metal halide perovskite is promising for highly efficient and thermally stable perovskite light-emitting diodes (PeLEDs). However, there is still great room for improvement in the film quality, including low coverage and high trap density, which play a vital role in achieving high-efficiency PeLEDs. In this work, lead acetate (Pb(Ac)_2_) was introduced into the perovskite precursor solution as an additive. Experimental results show that perovskite films deposited from a one-step anti-solvent free solution process with increased surface coverage and reduced trap density were obtained, leading to enhanced photoluminescence (PL) intensity. More than that, the valence band maximum (VBM) of perovskite films was reduced, bringing about a better energy level matching the work function of the hole-injection layer (HIL) poly (3,4-ethylenedioxythiophene)-poly (styrene sulfonate) (PEDOT: PSS), which is facilitated for the hole injection, leading to a decrease in the turn-on voltage (V_th_) of PeLEDs from 3.4 V for the control device to 2.6 V. Finally, the external quantum efficiency (EQE) of the sky blue PeLEDs (at 484 nm) increased from 0.09% to 0.66%. The principles of Pb(Ac)_2_ were thoroughly investigated by using X-ray photoelectron spectroscopy (XPS) and Fourier transform infrared spectroscopy (FTIR). This work provides a simple and effective strategy for improving the morphology of perovskite and therefore the performance of PeLEDs.

## 1. Introduction

Since the first report of halide perovskite light-emitting diodes (PeLEDs) in 2014 [1], great attention has been garnered due to the unique properties of halide perovskite, such as high photoluminescence quantum yield (PLQY), adjustable bandgap, excellent charge carrier mobility, and simple deposition process [2,3,4]. In the past decade, the performance of PeLEDs has made great progress, with the external quantum efficiency (EQE) of green, red, and blue PeLEDs exceeding 25% [5], 30% [6], and 15% [7], respectively, exhibiting broad application potential for solid-state-lighting and flat-panel displays.

In particular, full-inorganic metal halide perovskites (MHPs) are more thermal stable than the organic–>inorganic hybrid counterpart, providing a bright future as the emissive layer (EML) of PeLEDs [8]. A solution-based processing technique was widely used to deposit perovskite film due to its simplicity and compatibility with flexible substrates [9]. However, the MHP film usually suffers from poor morphology with low surface coverage on substrates, inducing a large quantity of defects, which is a disadvantage for the performance of PeLEDs [10]. It was mainly caused by the low solubility of cesium halide in the solvent of the perovskite precursor solution, which leads to a lack of sufficient raw materials in the formation process of perovskite, resulting in the formation of discontinuous thin-film morphology [10]. For its application in perovskite solar cells as the light-absorbing layer, a multi-step deposition method was developed to achieve thick perovskite film involving multiple spin-coating of cesium halide solution [11]. As for the EML of PeLEDs, a continuous thin-film morphology rather than a thick film is the main requirement for achieving high luminous efficiency. Thus, improving the quality of MHP films prepared by the one-step method is a feasible way to obtain high-efficiency light-emitting diodes.

The anti-solvent washing method, additive engineering, and interface modification are commonly used strategies to improve the film quality of perovskite film [12,13,14], where additive engineering involves adding additives to the precursor solution of perovskite, which can control the crystallization of the perovskite film, passivate defects, and enhance the charge carrier mobility. For example, the materials of Lewis bases can provide non-bonded electron pairs to coordinate with uncoordinated Pb^2+^ or metal Pb clusters, which are the main point defects in perovskite films [13]. S-H, C=O, and P=O groups in organic materials have a strong coordination effect with Pb^2+^ [15]. Methylamine acetate (MAAc) ionic liquid was introduced into the precursor solution to modulate the crystallization kinetics of perovskite film through strong interaction between Ac^−^ and Pb^2+^ [16]. Among numerous passivating agents, lead acetate has attracted our attention [17]. With lead acetate, improved film quality of perovskite can be achieved without an anti-solvent process added during spin-coating of the perovskite precursor solution, avoiding issues such as the poor batch-to-batch repeatability of devices and the potential harm to the environment of using toxic solvents [10]. However, the application of lead acetate additives in perovskite EML and its effect on the performance of PeLEDs has rarely been studied, which is addressed in this work.

In this work, Pb(Ac)_2_ was added to the precursor solution of perovskite as an additive to improve the film quality of perovskite film. Full-inorganic perovskite with a mixture of Br and Cl was used to realize sky blue PeLEDs. Experimental results show that after the addition of Pb(Ac)_2_, perovskite films with significantly improved surface coverage and reduced crystal size were obtained, yielding an increase in photoluminescence (PL) intensity. Moreover, the valance band maximum (VBM) of perovskite was lifted from −6.58 eV to −5.6 eV, leading to a reduction in the hole injection barrier because of the better energy level matching with the work function of hole injection layer (HIL) poly(3,4-ethylenedioxythiophene)-poly(styrenesulfonate) (PEDOT:PSS). Finally, the EQE of sky blue PeLEDs was greatly increased from 0.09% to 0.66%. A thorough investigation was conducted into the reasons for the performance improvement. This work provides an effective and convenient method to improve the efficiency of all-inorganic sky blue PeLEDs.

## 2. Results

### 2.1. The Morphology and Film Quality of Perovskite Film

**Figure 1:**
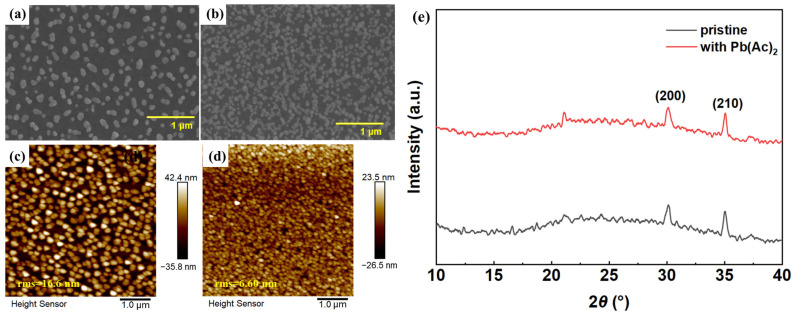
SEM (**a**,**b**) and AFM (**c**,**d**) images of perovskite films without and with Pb(Ac)_2_: (**a**,**c**) pristine; (**b**,**d**) with Pb(Ac)_2_; (**e**) XRD patterns of perovskite films.

Scanning electron microscopy (SEM) and atomic force microscopy (AFM) were used to inspect the effect of Pb(Ac)_2_ on the morphology of the perovskite layer, as shown in Figure 1. The pristine perovskite film shows poor coverage on the substrate with a large number of pinholes. With the introduction of Pb(Ac)_2_, the surface coverage of perovskite film on the substrate was significantly improved, and the grain size was reduced. AFM images also show the improved surface coverage and reduced crystal size of the perovskite film, which is consistent with the SEM images. In addition, the surface roughness of the perovskite film decreased greatly, with the root mean square (RMS) values reduced from 16.6 nm for the pristine film to 6.60 nm for the film with Pb(Ac)_2_, which is favorable for the deposition of 1,3,5,-tris(1-phenyl-1H-benzimidazol-2-yl)benzene (TPBi) on top of it. X-ray diffraction (XRD) patterns were also collected, which have been commonly used to study the crystal structure and orientation of perovskite films. The two distinguishing peaks at 30° and 35° correspond to the (200) and (210) crystal planes of the perovskite, respectively. After the introduction of Pb(Ac)_2_, the intensity of the XRD peaks decreased a little from 142 to 104 (at 30°) with the peak position unchanged, indicating a decrease in crystal size [18].

**Figure 2:**
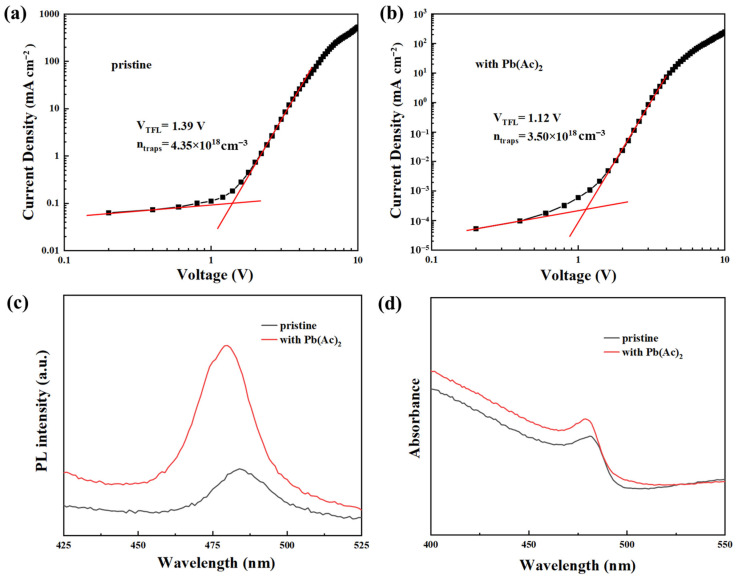
J-V curves of hole-only devices: (**a**) pristine; (**b**) with Pb(Ac)_2_; (**c**) PL; and (**d**) UV-Vis absorption spectra of perovskite films.

Morphology improvement will result in a decrease in defects in the perovskite film. Space-charge-limited current (SCLC) measurements have usually been used to estimate the defect density in perovskite films by analyzing the J-V of hole-only devices, which is shown in Figure 2. The knee point voltage from the linear ohmic region to the trap-limited current region is called the trap-filling voltage (*V_TFL_*), which is connected to the *N_defect_* according to Equation (1) [19].

(1) $$N_{defect}=\frac{2\varepsilon \varepsilon_{0}V_{TFL}}{eL^{2}}$$

where *ε* is the relative dielectric constant (*ε_perovskite_* = 34.6) [20], *ε*_0_ is the vacuum dielectric constant, *L* is the thickness of the perovskite film, and *e* is the elementary charge. The *V_TFL_* values obtained from the J-V curve of the control device and device with Pb(Ac)_2_ were 1.39 V and 1.12 V, respectively. The calculated *N_defect_* values were 4.35 ± 0.02 × 10^18^ cm^−3^ and 3.50 ± 0.02 × 10^18^ cm^−3^, respectively, indicating a better film quality of perovskite film with reduced *N_defect_* after the introduction of Pb(Ac)_2_. The error value was calculated by subtracting the average value and then divided by two. The J-V curves and the corresponding defect density values are shown in Figure S1 and Table S1.

The morphology of perovskite film has a vital influence on its optical properties, which was verified by measuring PL and UV-Vis absorption spectra of perovskite film with and without Pb(Ac)_2_, as shown in Figure 2c,d. PL spectra of both perovskite films showed similar shapes, while the PL intensity of the perovskite film with Pb(Ac)_2_ is much higher than that of the pristine film, suggesting a more efficient radiative recombination, which can be ascribed to the decrease in defect density [21,22]. Moreover, it is noticed that the peak of PL spectra blueshifted 4 nm from 484 nm for the pristine film to 480 nm for the film with Pb(Ac)_2_. For the UV-Vis absorption spectra of perovskite films, stronger light harvesting was observed with the addition of Pb(Ac)_2_, which benefited from the morphology improvement with improved surface coverage and reduced trap density. Similarly, a blueshift of 2 nm for the absorption peak was demonstrated, which moved from 481 nm to 479 nm after the addition of Pb(Ac)_2_. This is consistent with the blueshift of the PL spectra, which can be ascribed to the quantum confinement effect because of the reduced crystal size of the perovskite film [23,24].

**Figure 3:**
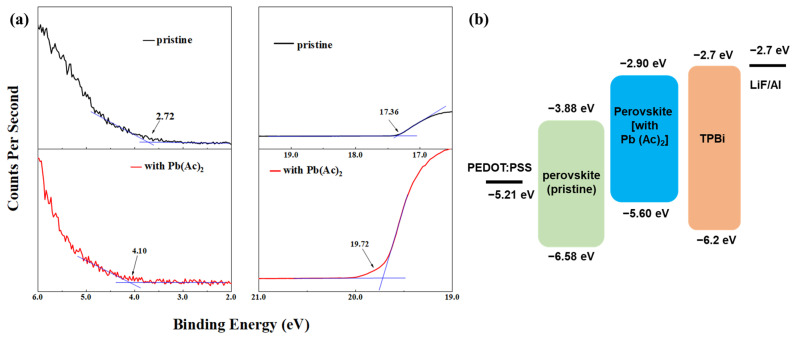
(**a**) UPS spectra of the perovskite films with and without Pb(Ac)_2_ and (**b**) the corresponding energy level alignment.

The electronic energy structure of the perovskite films with or without Pb(Ac)_2_ was also characterized by using ultraviolet photoelectron spectroscopy (UPS), as shown in Figure 3. Secondary-electron cutoff (E_cutoff_) at the high binding energy region and the onset energy (E_onset_) values were extracted from the UPS spectra in Figure 3a. Accordingly, the VBM values can be estimated (VBM = 21.22 eV − (E_cutoff_ − E_onset_)), which are −6.58 eV and −5.60 eV for the perovskite film without and with Pb(Ac)_2_, respectively. The corresponding energy level alignment between the perovskite film and the functional layers is depicted in Figure 3b. The shallower VBM of perovskite promotes better energy level alignment with m-PEDOT:PSS. It is advantageous for the injection of the hole from m-PEDOT:PSS to perovskite, which will lead to a decrease in the threshold voltage (V_th_).

### 2.2. The Interaction between Pb(Ac)_2_ and Perovskite

**Figure 4:**
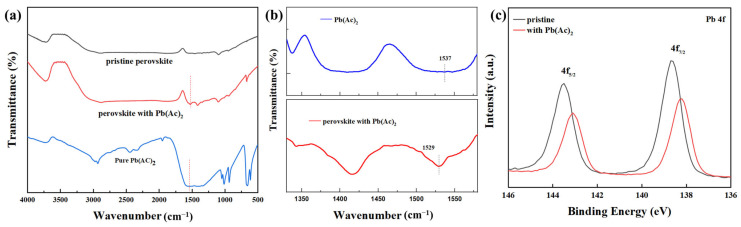
(**a**) FTIR spectra of perovskites and pure Pb(Ac)_2_, (**b**) the partially enlarged FTIR spectra of pure Pb(Ac)_2_ and perovskite with Pb(Ac)_2_, (**c**) XPS spectra of the core level of Pb 4f of the perovskite films without and with Pb(Ac)_2_.

To investigate the interaction between Pb(Ac)_2_ and perovskite, X-ray photoelectron spectroscopy (XPS) and Fourier transform infrared spectroscopy (FTIR) spectra were collected, as shown in Figure 4. In the FTIR spectra, the peak at 1537 cm^−1^ for pure Pb(Ac)_2_ was assigned to the stretching vibration of the COO^−^ (Ac^−^) anion, which moved to 1529 cm^−1^ for the perovskite with Pb(Ac)_2_. It implied a strong coordination interaction between the Pb^2+^ in the perovskite and the Ac^−^ in the Pb(Ac)_2_ by Lewis acid–base adduction [25]. With Pb(Ac)_2_, the XPS spectra of the core level of Pb 4f_7/2_ and Pb 4f_5/2_ shifted from 143.5 eV and 138.7 eV for the pristine perovskite film to 143.1 eV and 138.2 eV, respectively. The downshift in the core level of Pb 4f indicated that the chemical environment of Pb has changed [26,27], which induced the shift in the core levels of Br 3d, Cs 3d, and Cl 2p. The XPS full spectra and the spectra of the core levels of Br 3d, Cs 3d, and Cl 2p are shown in Supplementary Figure S2. Because of the strong interaction between the Pb^2+^ in the perovskite and the Ac^−^ in the Pb(Ac)_2_, the crystal growth rate of the thin films was slowed down [28], giving rise to the improved morphology of the perovskite with a higher surface coverage. In addition, the composition of perovskite film was verified by using semi-quantitative elemental analysis with XPS. The elemental contents of Cs, Pb, Br, and Cl of the pristine perovskite film are summarized in Table S2. It shows that the mole ratio of Br and Cl was about 2:1, indicating that the component of the perovskite is CsPb(Br_0.67_Cl_0.33_)_3_.

### 2.3. Device Performance

**Figure 5:**
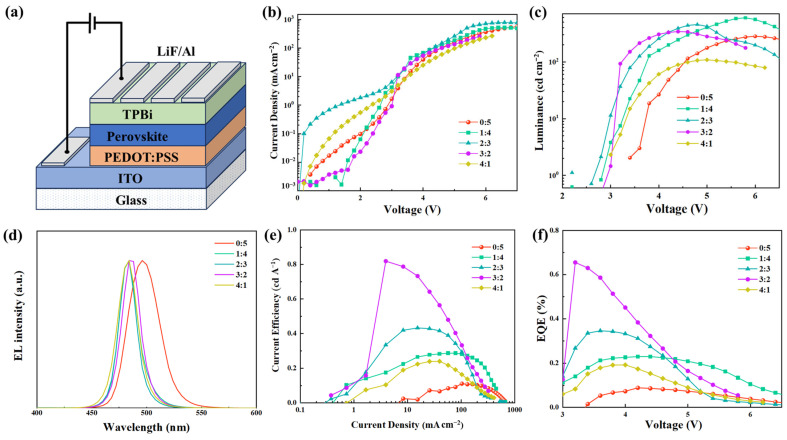
(**a**) Diagram of device structure; (**b**) current density–voltage curves (**c**) luminance–voltage curves; (**d**) electroluminescence spectra; (**e**) current efficiency–current density curves; and (**f**) external quantum efficiency–voltage curves of PeLEDs based on perovskite film with different ratios of Pb(Ac)_2_ in the precursor solution.

The effect of Pb(Ac)_2_ on device performance was studied by fabricating PeLEDs with structures of indium tin oxide (ITO)/m-PEDOT: PSS (50 nm)/CsPb(Br_0.67_Cl_0.33_)_3_ (35 nm)/TPBi (40 nm)/lithium fluoride (LiF) (1 nm)/Al (120 nm), where m-PEDOT:PSS is poly(3,4-ethylene dioxythiophene):poly(styrene sulfonate) (PEDOT:PSS) doped with poly(sodium 4-styrene sulfonate). The diagram of the device structure is depicted in Figure 5a. The J-V, L-V, LE-J, and EQE-V curves of the PeLEDs were collected and are shown in Figure 5. The corresponding detailed device performance parameters are summarized in Table 1. Figure 5 and Table 1 show that the EQE of the PeLEDs exhibits a parabolic variation trend as the mole ratio of Pb(Ac)_2_ to PbBr_2_ increased from 0:5, 1:4, 2:3, 3:2 to 4:1. With the ratio of 3:2, the PeLEDs had the highest EQE of 0.66%, which is much higher than that of the control device (0.09%). Meanwhile, the V_th_ of the PeLEDs decreased from 3.4 V for the control device to 2.6 V after the introduction of Pb(Ac)_2_, which can be ascribed to the reduced hole injection energy barrier. In addition, the EL peak (Figure 5d) of the control device was located at 496 nm, which moved to 484 nm with Pb(Ac)_2_. It agrees with the blueshift of the PL and UV-Vis spectra after the introduction of Pb(Ac)_2_. We noticed that for the control device, the EL spectrum shows a redshift of 12 nm compared to the corresponding PL spectrum, which is 4 nm for the device with Pb(Ac)_2_. The redshift of the EL peak relative to the PL can be ascribed to the interaction between the charge carrier and the exciton dynamics, which is related to the transition of the injected carriers to lower energy sites through the thermal relaxation process [29]. The corresponding Commission Internationale d’Eclairage (CIE) coordinates of the PeLEDs with and without Pb(Ac)_2_ are listed in Table 1.

**Figure 6:**
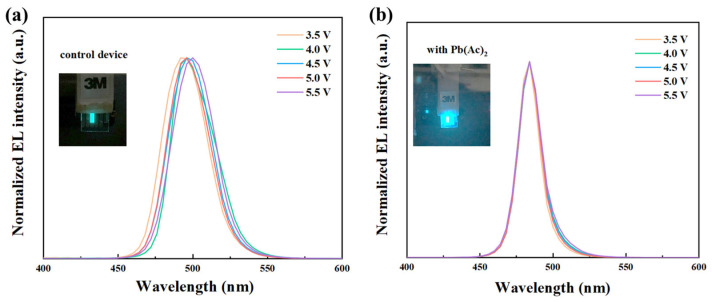
EL spectra of PeLEDs under different forward bias: (**a**) control and (**b**) with Pb(Ac)_2_. Inset: photographs of PeLEDs at a voltage of 4.5 V.

The voltage stability of the EL spectra of the PeLEDs with and without Pb(Ac)_2_ was recorded under bias voltage from 3.5 V to 5.5 V, as shown in Figure 6. With the increase in voltage, the EL peak of the control device moved from 492 nm and 496 nm to 500 nm at 5.5 V. It is obvious that the control device presents unstable spectra under different voltages, while the EL spectra of the Pb(Ac)_2_-based device stabilized at 484 nm with the voltage increase from 3.5 V to 5.5 V, demonstrating improved voltage stability for the EL spectra of the PeLEDs. The interaction between the Pb^2+^ in the perovskite and the Ac^−^ in the Pb(Ac)_2_ passivated the uncoordinated Pb^2+^ during crystallization progress, which effectively inhibited the migration of halide ions [23]. As a result, the voltage stability of the EL spectra of the PeLEDs was significantly improved. In addition, the operational stability of the control device and the device with Pb(Ac)_2_ were tested under constant current at 2 mA, as shown in Supplementary Figure S3. It showed that the half-efficiency lifetime of the control device is 19 s, which is 21 s for the device with Pb(Ac)_2_, indicating a slightly improved operational stability.

## 3. Discussion

After the addition of lead acetate in the precursor solution of perovskite, not only were the coverage of the perovskite films on the substrate significantly improved and the defect density of the perovskite film reduced, but the VBM of the perovskite film was also reduced. Thus, the PL luminescence intensity and the absorbance of the perovskite films are enhanced, which is ascribed to the film quality improvement of the perovskite films. A better energy level alignment between the VBM of the perovskite and the work function of the m-PEDOT: PSS was achieved. At the same time, the reduction in perovskite grains caused a slight blueshift in the PL and EL spectra. XPS and FTIR experiments were conducted to investigate the reasons for the improvement of film quality. The results showed that after adding lead acetate, the core levels of Pb 4f_7/2_ and Pb 4f_5/2_ shifted towards lower binding energy, and the vibrational energy level of the Ac^–^ anion shifted towards lower wavenumbers, indicating that the Ac^–^ in lead acetate interacted with the lead in the perovskite. The device performance shows that after using lead acetate, the EQE values increased from 0.09% to 0.66%, and the turn-on voltage of the device decreased from 3.4 V to 2.6 V.

The addition of lead acetate significantly improves the coverage of perovskite films on the substrate, which improves film quality, and thus enhances device efficiency. However, there is still large room for improvement in the quality of perovskite film, in which the defect density is relatively high. In future work, some strategies will be combined to control the crystallization kinetics of the perovskite films and improve the device efficiency. For example, using ligand molecules with multi-dentate functional groups as additives will increase the formation energy of halide defects and reduce the defect density, resulting in excellent perovskite layers and thereby improved device performance. Reducing the charge carrier injection barrier will balance carrier injection and therefore improve the luminescence efficiency of the PeLEDs.

## 4. Materials and Methods

### 4.1. Materials

Most of the materials used in this work were purchased from Xi’an Polymer Light Technology Corp (Xi’an, China), including Pb(Ac)_2_ (99.99%), cesium bromide (CsBr, 99.99%), lead bromide (PbBr_2_, 99.99%), lead chloride (PbCl_2_, 99.99%), TPBi, ITO, and PEDOT: PSS (Clevios™ P VP AI 4083). Sigma-Aldrich (St. Louis, MO, USA) provided us with poly(sodium 4-styrene sulfonate), polyethylene glycol (PEG), dimethylsulfoxide (DMSO), and LiF. All materials were utilized as is without additional purification steps.

### 4.2. Solution Preparation

The CsPb(Br_0.67_Cl_0.33_)_3_ precursor solution (0.12 mol/L) was prepared by co-dissolving CsBr, PbBr_2_, PbCl_2_, and Pb(Ac)_2_ in DMSO under continuous stirring overnight at room temperature. Among them, the mole ratio of PbCl_2_ to the sum of Pb(Ac)_2_ and PbBr_2_ was fixed at 0.5, while the mole ratio of Pb(Ac)_2_ to PbBr_2_ was varied from 0:5, 1:4, 2:3, 3:2 to 4:1 to optimize the content of Pb(Ac)_2_. The composition of CsBr was adjusted to maintain a specific bromine–chlorine ratio of 2:1. PEG was dissolved in DMSO at a precise concentration of 10 mg/mL before being incorporated into the perovskite precursor solution at a specified weight ratio of 80%. The m-PEDOT:PSS solution was prepared by combining a pure PEDOT:PSS aqueous solution with poly(sodium 4-styrene sulfonate) at a certain volume ratio of 3:1.

### 4.3. Device Fabrication

Device structure of ITO/m-PEDOT:PSS (50 nm)/CsPb(Br_0.67_Cl_0.33_)_3_ (35 nm)/TPBi (40 nm)/LiF (1 nm)/Al (120 nm) were used to fabricate PeLEDs. Before using the ultrasonic treatment in detergent, acetone, deionized water, and isopropanol were applied to thoroughly clean the ITO glass, following a 30 min drying process in an oven set at 80 °C. The ITO substrates underwent an additional step of O_2_ plasma treatment lasting 3 min to eliminate any remaining organic contaminants and enhance the ITO’s work function. The m-PEDOT:PSS solution was passed through a 0.22 µm water-based filter before being spin-coated onto the freshly cleaned ITO-coated glass at 7000 rpm for 60 s to produce a 50 nm thick m-PEDOT:PSS film. Afterward, the sample was placed on the hotplate and baked at 160 °C for 20 min. Following, the substrates were moved into an N_2_ glovebox with low water and oxygen content (both less than 1 ppm). The perovskite precursor solution was then spin-coated onto the m-PEDOT:PSS surface at 8000 rpm for 60 s, resulting in a 35 nm thick film. It was subsequently subjected to a heat treatment at a temperature of 80 °C for 20 min. Lastly, TPBi, LiF, and Al layers, measuring 40 nm, 1 nm, and 120 nm in thickness respectively, were sequentially deposited through thermal processes in a vacuum pressure of 4 × 10^−4^ Pa. The formation of a 0.1 cm^2^ active region was achieved by employing a cathode mask.

### 4.4. Measurement and Characterization

The morphology of perovskite films was observed through field emission scanning electron microscopy (FESEM, S4800 microscope, Hitachi Ltd., Tokyo, Japan). The study, conducted with an atomic force microscope (AFM, Bruker Dimension^®^ Icon™, Bruker Corporation, Berlin, Germany), involved analyzing the surface features of HTL and perovskite film under room temperature conditions. We collected X-ray diffraction (XRD) patterns of the perovskite films on a Bruker D8 ADVANCE X-ray diffractometer (Bruker Corporation, Germany) with operating conditions set at 40 kV and 40 mA. Analysis of X-ray photoelectron spectroscopy (XPS) and ultraviolet photoelectron spectroscopy (UPS) was conducted with a KRATOS Axis Supra (Kratos Analytical Ltd., Manchester, UK). Fourier transform infrared spectroscopy (FTIR) spectra were obtained with a Shimadzu IRPrestige-21 spectrophotometer (Shimadzu, Kyoto, Japan). We conducted absorption testing on a Lamba 35 spectrophotometer (Perkin-Elmer, Waltham, MA, USA). An FLSP920 spectrometer, manufactured by Edinburgh Instruments Ltd. in Livingston, UK, was utilized to collect the PL spectra. Utilizing a Keithley 2400 source measurement unit, a Keithley 2000 digital multimeter (Keithley, Solon, OH, USA), and a calibrated silicon photodiode, data for current density–operation voltage (J-V), luminous efficiency–current density (LE-J), external quantum efficiency–voltage (EQE-V) and luminance–voltage (L-V) were acquired. The electroluminescent (EL) spectra were gathered with the help of a PR-655 SpectraScan Spectrophotometer (Photo Research, JADAK, Syracuse, NY, USA). A Bruker DektakXT Stylus Profiler (Bruker, Berlin, Germany) was used to measure the thicknesses of the m-PEDOT:PSS and perovskite films. The measurements referenced above were conducted in the atmosphere, and the devices were left unencapsulated.

### 4.5. Preparation for Characterization

Perovskite films for XRD, SEM, UV, PL, UPS, and XPS tests were all prepared by spin-coating perovskite precursor solution on ITO/m-PEDOT:PSS substrate with a concentration of 0.12 mol/L. It was spin-coated at 8000 rpm for 60 s. Then, the films were thermal annealed on a hot stage at 80 °C for 20 min. For FT-IR measurement, the above-mentioned perovskite films were scraped from ITO substrates and then blended with spectrum-grade KBr. The mixed powder was pressed into pieces before use.

## 5. Conclusions

In summary, by introducing Pb(Ac)_2_ into the perovskite precursor solution as an additive, the morphology of perovskite film was promoted with larger surface coverage and reduced defect density. XPS and FTIR spectra of perovskites demonstrated the interaction between the Pb^2+^ in the perovskite and the Ac^−^ in the Pb(Ac)_2_, which delayed the crystal growth rate of the perovskite film. Moreover, because of the lifted VBM of the perovskite, the energy level alignment between the perovskite and m-PEDOT:PSS was boosted. An EQE enhancement from 0.09% to 0.66% was achieved. Finally, a stable 484 nm sky blue EL emission was obtained. Meanwhile, the voltage stability of the EL spectra of the PeLEDs was optimized. This work provides a simple and effective method to improve the morphology of perovskite film and the performance of PeLEDs.

## Figures and Tables

**Table 1:** Detailed performance parameters of champion PeLEDs based on different mole ratios of Pb(Ac)_2_:PbBr_2_.

Pb(AC)_2_:PbBr_2_ (Mole Ratio)	V_th_ (V)	Max. L ^a^ (cd m^−2^)	Max. LE ^b^ (cd A^−1^)	Max. EQE ^c^ (%)	EL Peak (nm)	CIE
0:5	3.4	281.92	0.11	0.09	496	(0.065, 0.447)
1:4	2.8	597.17	0.29	0.23	484	(0.086, 0.209)
2:3	2.6	454.55	0.43	0.35	484	(0.090, 0.181)
3:2	2.8	341.79	0.82	0.66	484	(0.080, 0.224)
4:1	3.0	108.62	0.24	0.19	484	(0.093, 0.178)

^a^ Maximum luminance; ^b^ maximum current efficiency; ^c^ maximum external quantum efficiency.

## Data Availability

Data are contained within the article and Supplementary Materials.

## Supplementary Materials

The following supporting information can be downloaded at: https://www.mdpi.com/article/10.3390/molecules29112425/s1, Figure S1: The J-V curves of hole-only devices: (a) control device and (b) with Pb(Ac)_2_; Figure S2: XPS spectra of perovskite films: (a) full spectra, (b) Cs 3d, (c) Cl 2p, and (d) Br 3d.; Figure S3: The operational stability of control device and device with Pb(Ac)_2_.; Table S1: The statistical defect density values estimated from SCLC data. Table S2: Summary of the elemental content of Cs, Pb, Br, and Cl in the pristine perovskite film by using semi-quantitative elemental analysis with XPS.
