# Supplementary material for: Enhanced Efficiency and Stability of Sky Blue Perovskite Light-Emitting Diodes via Introducing Lead Acetate
Source: Molecules. 2024 May 21;29(11):2425. doi: 10.3390/molecules29112425 (PMC11174098; doi:10.3390/molecules29112425)
Supplement: Supplementary file 1 [file molecules-29-02425-s001.zip › molecules-2993187-supplementary.pdf]

Enhanced Efficiency and Stability of Sky Blue Perovskite Light−Emitting Diodes via Introducing Lead Acetate

Zequan Zhang [1], Qiaoli Niu [1,*], Baoxiang Chai [1], Junhao Xiong [1], Yuqing Chen [1], Wenjin Zeng [1], Xinwen Peng [2], Emmanuel Iheanyichukwu Iwuoha [3] and Ruidong Xia [1,*]

[1] State Key Laboratory of Organic Electronics and Information Displays & Institute of Advanced Materials (IAM), Nanjing University of Posts & Telecommunications, 9 Wenyuan Road, Nanjing 210023, China; zzq15235813322@163.com (Z.Z.); 18300633693@163.com (B.C.); 18790401377@163.com (J.X.); 1023061507@njupt.edu.cn (Y.C.); iamwjzeng@njupt.edu.cn (W.Z.)

[2] State Key Laboratory of Pulp and Paper Engineering, School of Light Industry and Engineering, South China University of Technology, Guangzhou 510640, China; fexwpeng@scut.edu.cn

[3] Sensor Lab (University of the Western Cape Sensor Laboratories), 4th Floor Chemical Sciences Building, University of the Western Cape, Robert Sobukwe Road, Bellville, Cape Town 7535, South Africa; eiwuoha@uwc.ac.za

* Correspondence: iamqlniu@njupt.edu.cn (Q.N.); iamrdxia@njupt.edu.cn (R.X

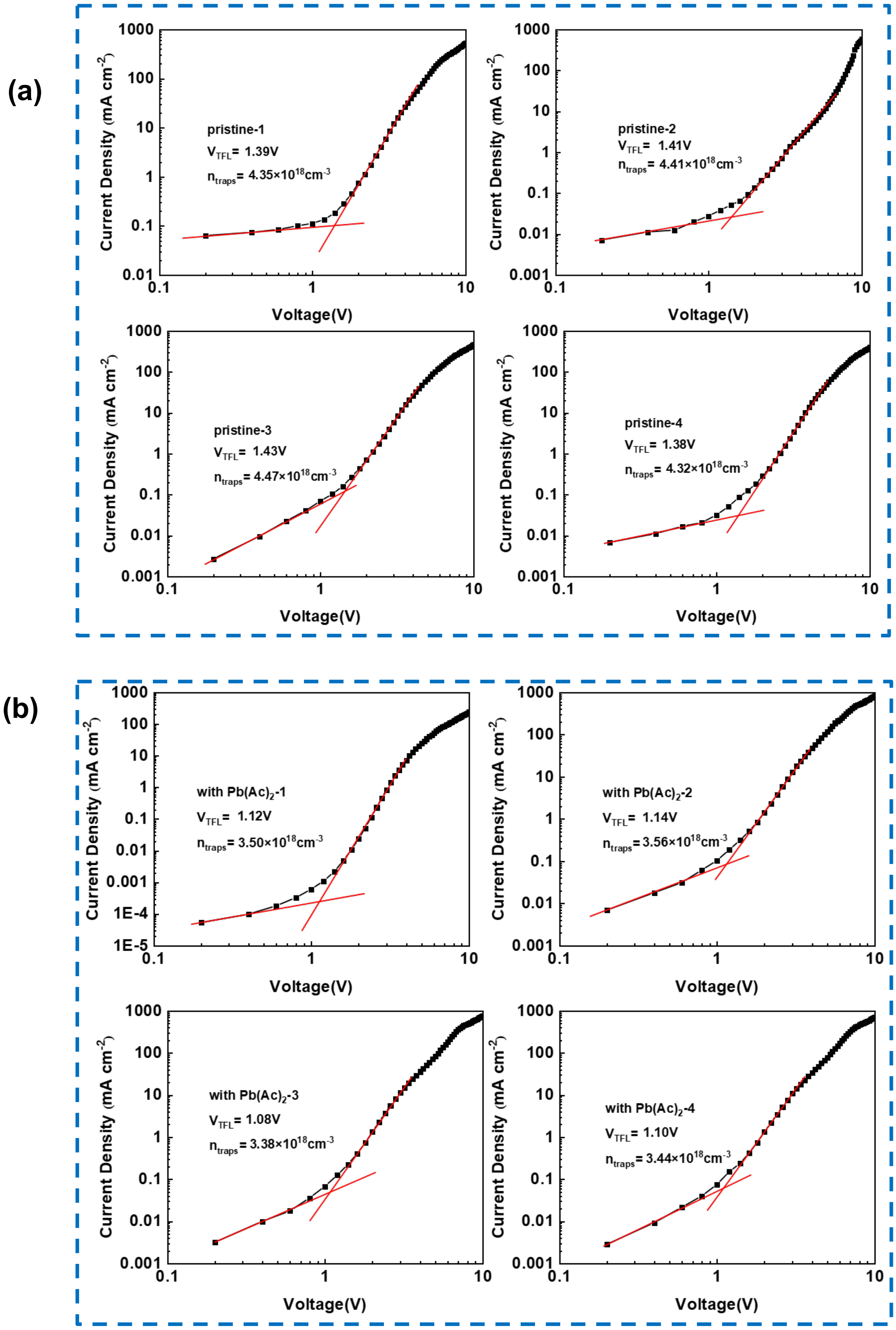

Figure S1. The J-V curves of hole-only devices: (a) control device, (b) with $Pb(Ac)_2$.

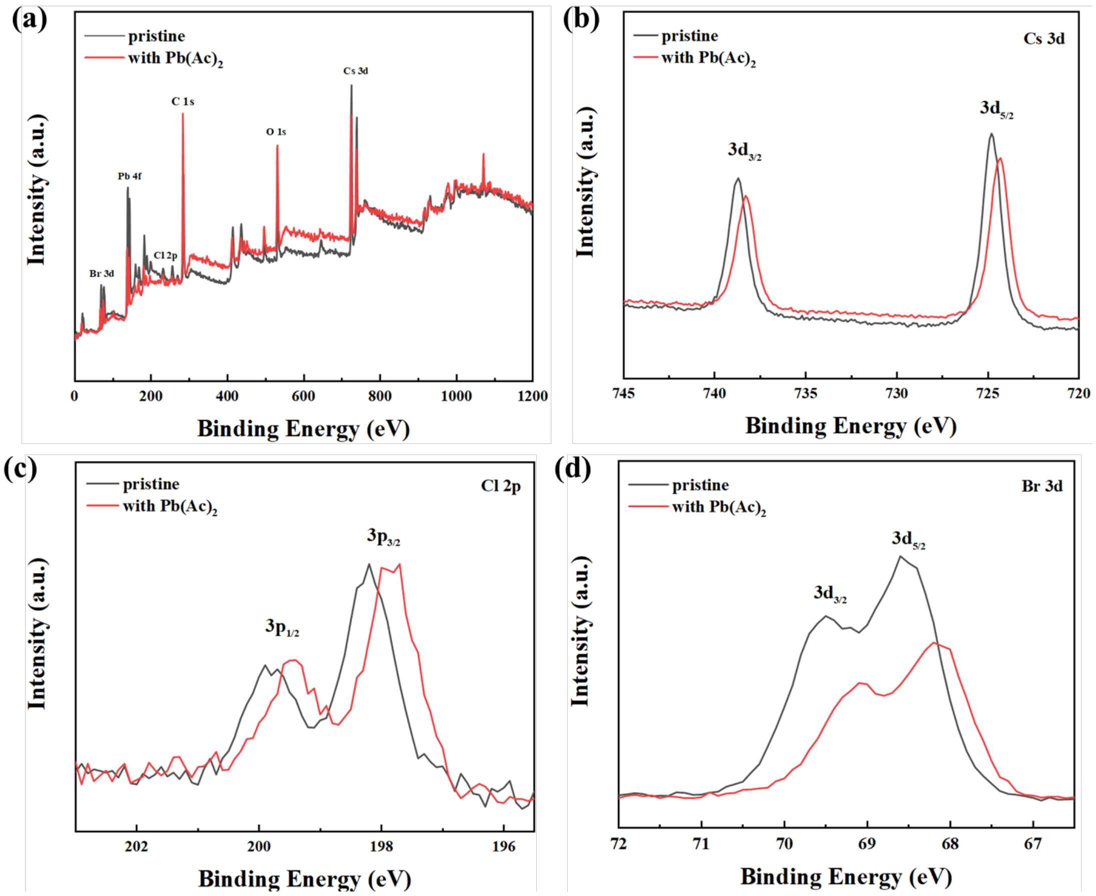

Figure S2. XPS spectra of perovskite films: (a) full spectra, (b) Cs 3d, (c) Cl 2p and (d) Br 3d.

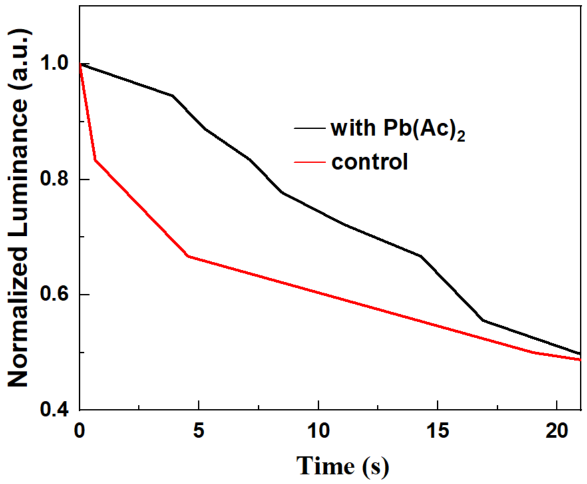

Figure S3. The operational stability of control device and device with $Pb(Ac)_2$.

Table S1. The statistical defect density values estimated from SCLC data.

	Data 1 ($\times10^{18}$ cm^{-3})	Data 2 ($\times10^{18}$ cm^{-3})	Data 3 ($\times10^{18}$ cm^{-3})	Data 4 ($\times10^{18}$ cm^{-3})	Average ($\times10^{18}$ cm^{-3})
control	4.35	4.41	4.47	4.32	4.39
with $Pb(Ac)_2$	3.5	3.56	3.38	3.44	3.47

Table S2. Summary of the elemental content of Cs, Pb, Br and Cl in the pristine perovskite film by using semi-quantitative elemental analysis with XPS.

element	FWHM (eV)	area (CPS·eV)	elemental content (at.%)
Cs3d	2.71	203109.8	21.93
Pb4f	1.64	265839	16.32
Br3d	2.79	55152.07	40
Cl2p	4.14	27939.94	21.74
